# Identification of a novel polyprenylated acylphloroglucinol-derived SIRT1 inhibitor with cancer-specific anti-proliferative and invasion-suppressing activities

**DOI:** 10.3892/ijo.2014.2639

**Published:** 2014-09-03

**Authors:** LIJIA ZHU, JI QI, CHRISTINE YA-CHI CHIAO, QIANG ZHANG, JOHN A. PORCO, DOUGLAS V. FALLER, YAN DAI

**Affiliations:** 1 Cancer Research Center and Department of Medicine, Boston University School of Medicine, Boston, MA 02118, USA; 2 Department of Chemistry, Center for Chemical Methodology and Library Development (CMLD-BU), Boston University, Boston, MA 02215, USA

**Keywords:** SIRT1 inhibitor, polyprenylated acylphloroglucinol, cancer cell growth, cancer cell invasion

## Abstract

SIRT1, a class III histone deacetylase, plays a critical role in regulating cancer cell growth, migration and invasion, which makes it a potential target for cancer therapeutics. In this study, we screened derivatives of several groups of natural products and identified a novel SIRT1 inhibitor JQ-101, a synthetic derivative of the polyprenylated acylphloroglucinol (PPAP) natural products, with an IC_50_ for SIRT1 of 30 μM *in vitro*, with 5-fold higher activity for SIRT1 vs. SIRT2. Exposure of tumor cells to JQ-101 significantly enhanced acetylation of p53 and histone H4K16 at known sites of SIRT1 deacetylation, validating SIRT1 as its cellular target. JQ-101 suppressed cancer cell growth and survival by targeting SIRT1, and also exhibited selective cytotoxicity towards a panel of human tumor cell lines, while producing no toxicity in two normal human cell types at comparable concentrations. JQ-101 induced both apoptosis and cell senescence, and suppressed cancer cell invasion *in vitro*. In summary, we have identified JQ-101 as a new SIRT1 inhibitor which may have potential application in cancer treatment through its ability to induce tumor cell apoptosis and senescence and suppress cancer cell invasion.

## Introduction

SIRT1 is a nicotinamide adenine dinucleotide (NAD)-dependent histone deacetylase that has been reported to play an important role in a variety of physiological processes including aging, metabolism, cell survival and carcinogenesis (1–6). Moreover, there is growing evidence strongly suggesting that SIRT1 has a role in cancer progression (7–14). SIRT1 suppresses apoptosis and cell senescence and also promotes cell migration and invasion (14–18), angiogenesis (19,20), and drug resistance in multiple models (7–10). *In vivo* studies show that SIRT1 inhibition suppresses tumor growth, metastasis and progression in several cancer types, including prostate, breast and neuroblastoma (11,12,14). In addition, SIRT1 overexpression has been shown to correlate with poor prognosis in several cancer types, including large B-cell lymphoma (21), prostate cancer (18,22), pancreatic cancer (23), gastric cancer (24,25), breast cancer (26), hepatocellular carcinoma (27), colorectal cancer (28) and lung cancer (29). Collectively, these results suggest an important role for SIRT1 in cancer growth and progression. SIRT1 inhibitors are therefore of significant interest as potential therapeutic agents.

Several inhibitors of SIRT1 have been reported, including nicotinamide (30), sirtinol (31), cambinol (32), EX-527 (33), Tenovin-6 (34), splitomycin (35), toxoflavin (36), salermide (37), 2-anilinobenzamides (38) among other compounds. These SIRT1 inhibitors can induce selective cytotoxicity in cancer cells *in vitro* (31,32,34–36,38,39). In addition, several SIRT1 inhibitors have been tested in cancer xenograft mouse models (32,34,40). Cambinol was well tolerated in mice and significantly inhibited the growth of Burkitt lymphoma xenografts (32). Tenovin-6 suppressed tumorigenesis of melanoma and N-Myc-induced neuroblastoma (34), and inauhzin, a phenothiazine, reduced colon xenograft growth (40). These results provide proof-of-concept examples that SIRT1 inhibition may be an effective modality in cancer therapy.

Here we report the identification of a new SIRT1 inhibitor, JQ-101, which induces cancer cell apoptosis and senescence, suppresses cancer cell invasion, and exerts cancer-specific cytotoxity, repressing tumor cell growth.

## Materials and methods

### Cells, antibodies and reagents

All cancer and normal cells lines were obtained from the American Type Culture Collection (Manassas, VA). LNCaP, PC3, Ramos, Jurkat, H1299 and MRC5 cells were maintained in RPMI-1640 medium with 10% FBS (HyClone, CO). H460, A549, ZR75 and MDA231 cells were maintained in DMEM medium with 10% FBS. PZ-HPV-7 cells were maintained in Keratinocyte Serum-Free Medium supplemented with Epidermal Growth Factor (Invitrogen, Carlsbad, CA).

Antibodies to SIRT1 (sc-74504) were purchased from Santa Cruz Biotechnology (Santa Cruz, CA). Antibodies to Ac-p53, p53, Ac-Histone H4 and H4 were purchased from Millipore (Billerica, MA). Antibodies to β-actin were purchased from Sigma-Aldrich (St. Louis, MO). Sirtinol was purchased from Sigma-Aldrich.

### Chemical synthesis of polyprenylated acylphloroglucinol (PPAP) analogues

**Figure 1:**
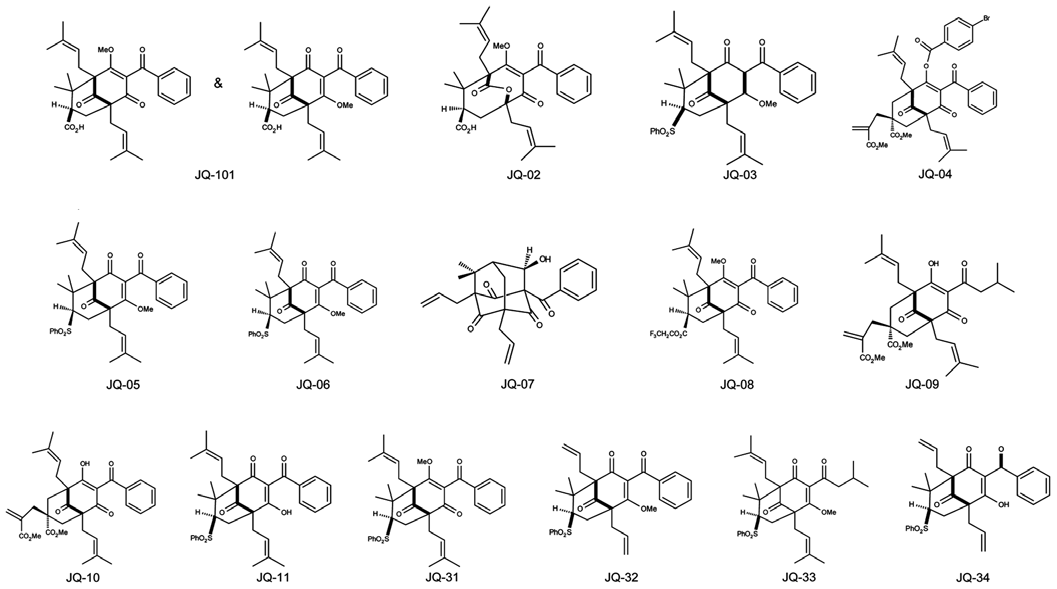

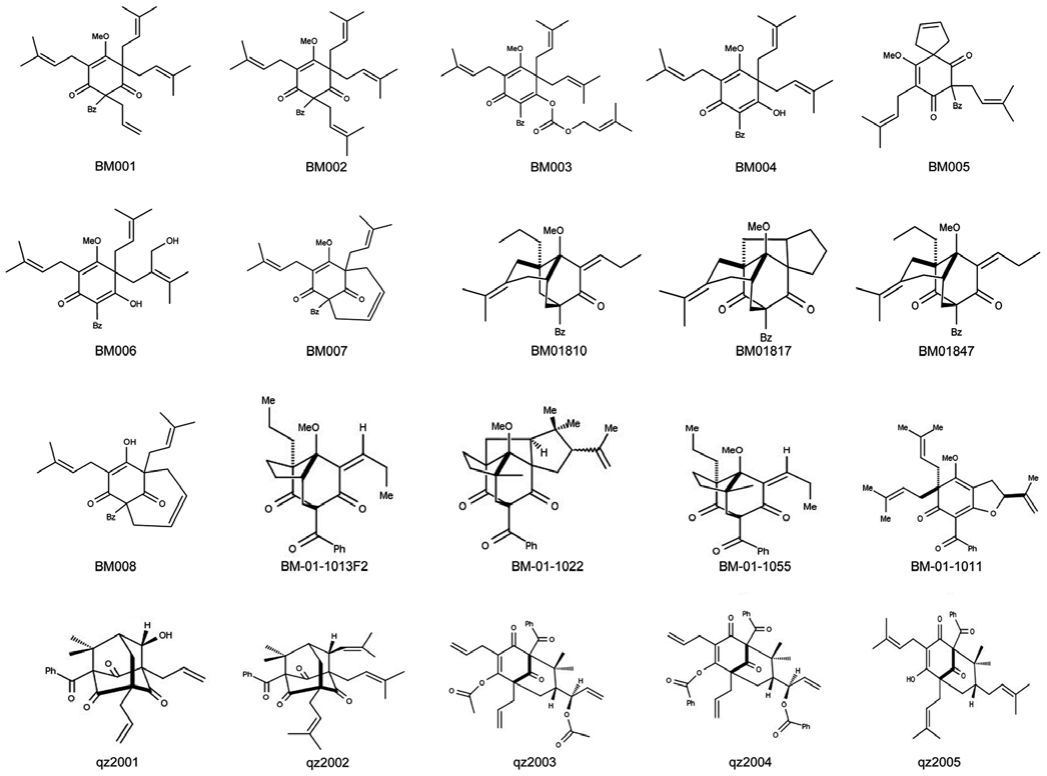
Synthesized and screened compounds. A panel of synthesized analogues of the type B PPAP natural product clusianone and the type A PPAP natural product nemorosone. The compounds were synthesized with a procedure involving tandem alkylative dearomatization/annulation of acylphloroglucinols or employing Mn(III)/Cu(II)-mediated oxidative radical cyclizations of dearomatized phloroglucinol substrates.

JQ-101, JQ-2, JQ-3, JQ-4, JQ-5, JQ-6, JQ-7, JQ-8, JQ-9, JQ-10, JQ-11, JQ-31, JQ-32, JQ-33 and JQ-34 (Fig. 1) are simplified analogues of the type B PPAP natural product clusianone and were synthesized using our reported procedure involving tandem alkylative dearomatization-annulation of acylphloroglucinols to rapidly construct the bicyclo[3.3.1] nonane-1,3,5-trione core (41). BM001, BM002, BM003, BM004, BM005, BM006, BM007, BM008, BM01810, BM01817, BM01847, BM-01-1005, BM-01-1013F2, BM-01-1011, BM-01-1022 and related bicyclo[2.2.2] octadiones (Table I) were synthesized using the reported method involving Mn(III)/Cu(II)-mediated oxidative radical cyclizations of dearomatized phloroglucinol substrates (42). Compounds QZ-2001-2005, analogues of the type A PPAP nemorosone, were prepared as intermediates during the course of our chemical synthesis of 7-epi-nemorosone (43).

### SIRT1 and SIRT2 activity analysis and small molecule screening

The Cayman hSIRT1 activity assay kit (SIRT1 Direct Fluorescent Screening Assay Kit, cat. no. 10010401) and hSIRT2 activity assay kit (SIRT2 Direct Fluorescent Screening Assay Kit, cat. no. 700280) were used to quantitate the IC_50_s of the SIRT inhibitors. The assay was carried out according to the manufacturer’s instructions. All compounds were preincubated with the hSIRT1/hSIRT2 proteins before commencing the reaction through the addition of the ‘Fluor de Lys’ deacetylase substrate. Deacetylation of K382-p53/K320-p53 was used as a marker of HDAC activity. Fluorescence was read (Ex 360 nm/Em 460 nm) using Synergy HT Multi-Mode Microplate Reader (BioTek). Assays were repeated in triplicate. Quantitation of acetylation was derived from the levels of fluorescence (displayed in arbitrary fluorescence units), and the percentages of inhibition were calculated from the arbitrary fluorescence unit of the treated assays and non-treated controls. The IC_50_ was determined using GraphPad Prism 6.0.

### Establishment of stable SIRT1-knockdown cell lines

The Phoenix packaging cell line was transfected with shRNA expression plasmids pSUPER.retro.puro-SIRT1 (5′-GATGAAGTTGACCTCCTCA-3′) or pSUPER.retro.puro-non-targeting shRNA control separately, using Lipofectamine 2000 (Invitrogen). After 48 h, the medium containing retrovirus was collected, filtered, treated with polybrene and transferred to LNCap or H460 cell cultures. Infected cells were selected with puromycin and the stably-infected colonies were pooled.

### Immunoblot analysis

Cells were lysed with 1% Nonidet P-40 lysis buffer (1% NP-40, 150 mM NaCl, 50 mM Tris-HCl) with protease inhibitor (Roche Diagnostics). Protein samples were subsequently separated on an 8% SDS-polyacrylamide gel and analyzed with anti-Ac-p53, anti-p53, anti-SIRT1, anti-Ac-Histone H4, anti-H4 or β-actin antibodies.

### Cell viability assay

MTS (Promega, G3580) assays were used to quantify cell viability. Cells were plated at a density of 10^4^ cells/well on 96-well plates and exposed to various concentrations of JQ-101, ranging from 5 to 125 μM, for 72 h. A total of 20 μl MTS CellTiter96Aqueous one solution reagent was added to the medium for 1 h, and the absorbance was read at 490 nm.

### Apoptosis analysis by FACS

The cultured cells were trypsinized, collected and labeled with Annexin V antibody and PI. The procedure for cell labeling followed the Annexin V kit instructions (Annexin V-PE Apoptosis Detection Kit I, BC Pharmingen, cat. no. 559763). The labeled samples were further analyzed with flow cytometry.

### SA-β-gal staining

The cells were seeded at 30% confluence, and JQ-101 was added to a final concentration 50 μM. At 4 days after the addition of JQ-101 to the culture media, the cells were washed with phosphate-buffered saline (PBS) and fixed with PBS containing 2% formaldehyde and 0.2% glutaraldehyde for 10 min. The cells were then incubated at 37°C overnight with staining solution [40 mM citric acid sodium phosphate, pH 6.0, 1 mg/ml 5-bromo-4-chloro-3-isolyl-β-D-galactoside (X-gal, Fisher, Pittsburgh, PA), 5 mM potassium ferricyanide, 150 mM NaCl, 2 mM MgCl_2_]. SA-β-gal-positive cells were enumerated by microscopy.

### Cell invasion assay

Cell invasion activity of cancer cells was assessed using BD BioCoat Matrigel Invasion Chambers (BD cat. no. 354480). Briefly, the cells were trypsined, washed with PBS, and resuspended in media containing 1% FBS at a density of 5×10^4^ cells/ml. A total of 500 μl of cells suspended in medium containing JQ-101 or DMSO (vehicle control) was placed in the upper chamber coated with matrigel and 500 μl of culture media was added to the lower chamber of the transwell. After 48 h of incubation at 37°C, the transwells were removed from the 24-well plates and stained with Diff-Quick solution. The invading cells were enumerated by microscopy.

## Results

### Design, synthesis and evaluation of SIRT1 inhibitors based on polyprenylated acylphloroglucinol (PPAP) natural products

Polyprenylated acylphloroglucinols (PPAPs) are a family of natural products that possess a wide range of different biological activities (44). There are more than 100 PPAPs discovered to date. Most of these were isolated from plants and trees from Clusiaceae (Guttiferae). PPAPs are divided into three types (A, B and C), depending on the position of the acyl group on the bicyclic core (44). It has been reported that the PPAPs guttiferone G and aristoforin have SIRT1 inhibitory activity, although little biological function has been shown (39). We synthesized a panel of derivatives of PPAP compounds using our reported procedure involving tandem alkylative dearomatization/annulation of acylphloroglucinols to rapidly construct the bicyclo[3.3.1] nonane-1,3,5-trione core (41) or employing Mn(III)/Cu(II)-mediated oxidative radical cyclizations of dearomatized phloroglucinol substrates (42).

**Figure 2:**
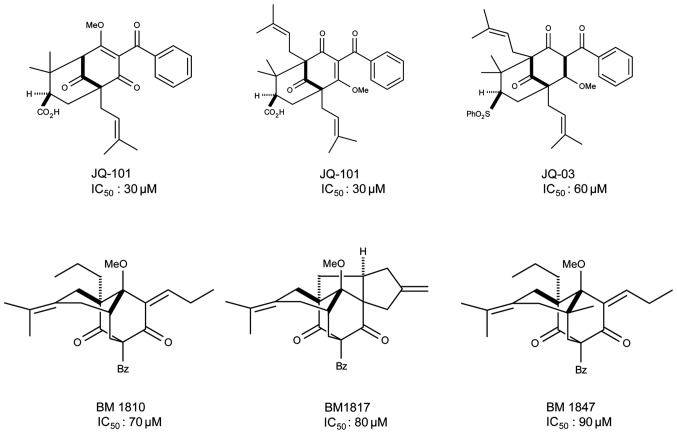
Compounds with SIRT1 inhibitory activity. A biochemical-based inhibitory assay with recombinant SIRT1 and SIRT2 was performed. Five compounds show SIRT1 inhibition activity with IC_50_ from 30 to 90 μM.

Using a fluorogenic substrate, we performed a biochemical-based inhibitory assay with recombinant SIRT1 and SIRT2. We synthesized and screened over 35 synthesized analogues of the type B PPAP natural product clusianone and the type A PPAP natural product nemorosone (see Fig. 1 for details and structures). Among the synthesized PPAP analogues, we identified five compounds with SIRT1 inhibitory activity with IC_50_ ranging from 30 to 90 μM, including JQ-101, JQ-3, BM1810, BM1817 and BM1847 (Fig. 2). One of these compounds, designated JQ-101, which showed the best *in vitro* activity for inhibition of SIRT1, forms the major focus of this report.

### Inhibition of SIRT1 deacetylase activity by JQ-101 in vitro and in vivo

**Figure 3:**
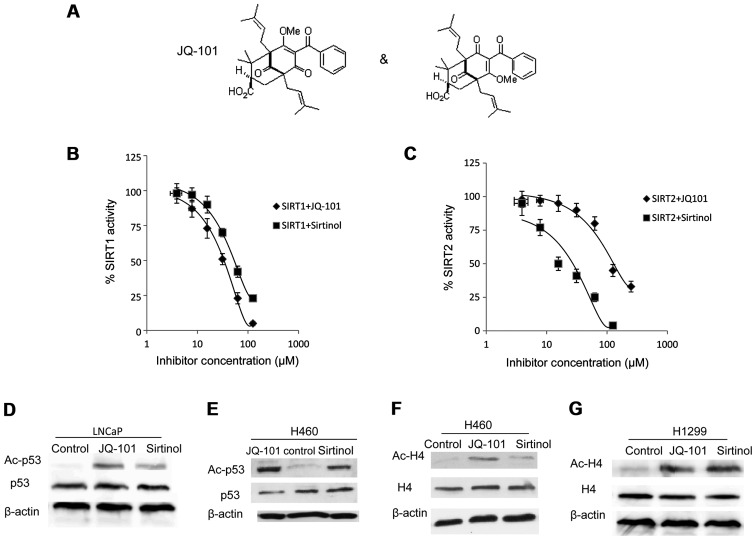
Identification of JQ-101 as a selective SIRT1 inhibitor. (A) Chemical structure of JQ-101. (B and C) *In vitro* SIRT1 and SIRT2 inhibition assays. Recombinant human SIRT1 and SIRT2 were exposed to various concentrations of JQ-101 and sirtinol as indicated, and their relative inhibitory potential (against DMSO as vehicle control) was analyzed and displayed as a percentage of SIRT1 activity. JQ-101 inhibited recombinant SIRT1 and SIRT2 deacetylase activity at an IC_50_ of 30 and 150 μM, respectively. Sirtinol was included as a positive control. The IC_50_ determination of JQ-101 was performed using GraphPad Prism 6.0. Each point represents the mean of three independent experiments; error bars indicate SD. (D and E) JQ-101 increases levels of lysine 382 acetylated p53. The human prostate cancer cell line (D) LNCaP and (E) H460 were treated with JQ-101 at 50 μM or sirtinol at 50 μM or vehicle control for 2 h. Cell lysates were prepared and immunoblotted with anti-Ac-p53, p53 or β-actin antibodies. (F and G) JQ-101 increased levels of histone H4 acetylated at lysine 16. (F) H460 and (G) H1299 cells were treated with JQ-101 at 50 μM or sirtinol at 50 μM or vehicle control for 2 h. Cell lysates were prepared and immunoblotted with anti-Ac-H4K16, anti-H4 or β-actin antibodies.

Using a fluorogenic substrate, we performed biochemical-based inhibition assays with recombinant SIRT1 and SIRT2. JQ-101 inhibited SIRT1 deacetylase activity with an IC_50_ of 30 μM (Fig. 3A and B). JQ-101 also inhibited the closely-related class III HDAC SIRT2, with an IC_50_ of 150 μM (Fig. 3C). Thus, JQ-101 has 5-fold selectivity in inhibiting SIRT1 over SIRT2. Sirtinol was used as a positive control for the assay, with an IC_50_ value of 60 μM for SIRT1 (Fig. 3B) and 20 μM for SIRT2 (Fig. 3C), respectively, in good agreement with reported values.

We next examined whether JQ-101 can target SIRT1 in a cell-based system. It is well established that lysine 382 of p53 is a cellular substrate of SIRT1 (46). We therefore assessed the levels of lysine 382-acetylated p53 in p53 wild-type cells exposed to JQ-101 compared to vehicle. Treatment with JQ-101 increased the acetylation levels of p53 in LNCaP and H460 cells (Fig. 3D and E). We also assessed the level of histone H4 acetylation at lysine 16, another confirmed substrate for SIRT1. JQ-101 treatment increased the levels of histone H4 acetylation in both H460 (p53 wt) and H1299 (p53 null) cells (Fig. 3E and F). These findings suggest that JQ-101 can prevent the deacetylation of known biological substrates of SIRT1, thereby demonstrating on-target SIRT1 inhibitory activity of JQ-101 *in vivo*.

### JQ-101 suppresses cancer cells growth

**Figure 4:**
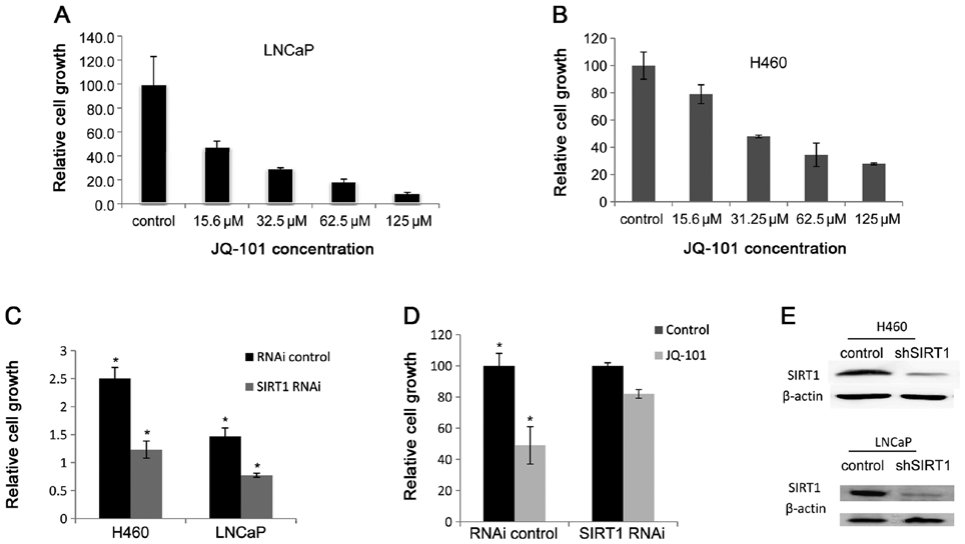
JQ-101 suppresses tumor cells growth and survival. (A and B) Cells from the prostate cancer line LNCaP or lung cancer line H460 were treated with varying concentrations of JQ-101 as indicated in the graph for 72 h, or vehicle control (DMSO) and MTS assay was performed. The error bars indicate the SEM. (C) SIRT1 silencing suppressed both LNCaP and H460 cell growth and survival. A total of 2×10^4^ SIRT-silenced or shRNA-control LNCaP or H460 cells were seeded onto 24-well plates and counted after 72 h of culture. The error bars indicate the SEM. (D) SIRT1 knockdown reduces the inhibitory effects of JQ-101 on H460 proliferation. A total of 2×10^4^ SIRT-silenced or shRNA-control H460 cells were seeded onto 24-well plates, treated with 50 μM JQ-101 for 3 days and viable cells were enumerated. The error bars indicate the SEM. Asterisks indicate significant differences between experimental versus control group (^*^p<0.05). (E) Immunoblot analysis shows the SIRT1 levels in the LNCap and H460 knockdown cells that were used for the cell proliferation analysis in (C and D).

Several SIRT1 inhibitors have been shown to produce antitumor activity *in vitro* and in mouse xenograft models. To investigate the effects of JQ-101 on cancer cell growth and survival inhibition, we determined the inhibitory ability of JQ-101 on a panel of human cancer cell lines, with normal human cell lines as serving as non-cancer controls. As shown in Fig. 4A and B, JQ-101 suppressed LNCaP and H460 tumor cell growth in a dose-dependent manner. We screened eight different cancer cell lines in total, including lines derived from prostate cancers (LNCaP and PC3), lung cancers (H460 and A549), leukemia (Jurkat), lymphoma (Ramos), and breast cancers (ZR75 and MDA231). JQ-101 suppressed tumor cell growth at IC_50_ values ranging from 20 to 119 μM (Table I). By comparison, JQ-101 was much less toxic in normal human cells, including one normal human prostate epithelial cell line (PZ-HPV-7) and one human lung fibroblast cell line (MRC-5) (Table I). These results suggest that JQ-101 exerts cancer cell-selectivity in inhibiting the growth of cells.

To more specifically study the role of SIRT1 in control of cancer cell growth, SIRT1 was knocked down with SIRT1 RNAi in LNCaP and H460 cells. SIRT1 silencing resulted in cancer cell growth suppression (Fig. 4C). This suggests that SIRT1 is important in control of growth in these cancer cells. To further study the specificity of JQ-101 mediated cancer cell growth, we treated the both SIRT1-silenced and vector control-transfected H460 cancer cell lines with JQ-101. The effect of JQ-101 on cancer cell growth is greatly attenuated in the SIRT1-silenced cells (Fig. 4D). This suggests that JQ-101 suppresses cancer cell growth by targeting SIRT1.

### JQ-101 induces both apoptosis and cell senescence

**Figure 5:**
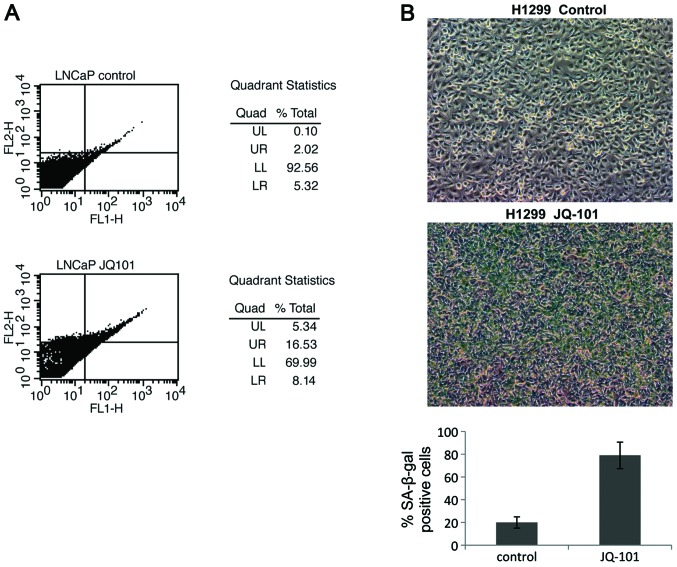
JQ-101 induces either apoptosis or cell senescence. (A) JQ-101 induces apoptosis in prostate LNCaP cells. LNCaP cells were treated with JQ-101 (50 μM) or vehicle control for 72 h. The cells were collected, labeled with Annexin V antibody/PI, and analyzed with flow cytometry. UL, dead cells; UR, late apoptosis; LL, live cells; LR, early apoptosis. (B) The lung cancer cell lines H460 was seeded at approximately 50% confluence and exposed to JQ-101 at 50 μM for 3 days. X-Gal-based β-galactosidase staining was performed. The β-galactosidase staining-positive cells were quantitated by microscopy and the percentage of SA-β-Gal positive cells was calculated. The histograms represent the mean ± SD of triplicates. Asterisks indicate significant differences between two groups (^*^p<0.01).

To further elucidate the molecular mechanisms of this tumor-selective growth suppression, Annexin V and β-gal staining were carried out to study the effects of JQ-101 in inducing apoptosis or cell senescence. JQ-101 treatment induced cell death and apoptosis in LNCaP cells; proportions of cells in both late stage (14.5%) and early stage (3.2%) apoptosis increased after JQ-101 treatment (Fig. 5A). In contrast, apoptosis was not observed in H1299 cells (data not shown); instead, JQ-101 treatment induced cell senescence in H1299 cells (Fig. 5B) but not in LNCaP cells (data not shown). This suggests that the induction of either apoptosis or cell senescence, in a context-dependent fashion, are the mechanisms whereby JQ-101 suppresses cancer cell growth and survival.

### JQ-101 inhibits cancer cell invasion

**Figure 6:**
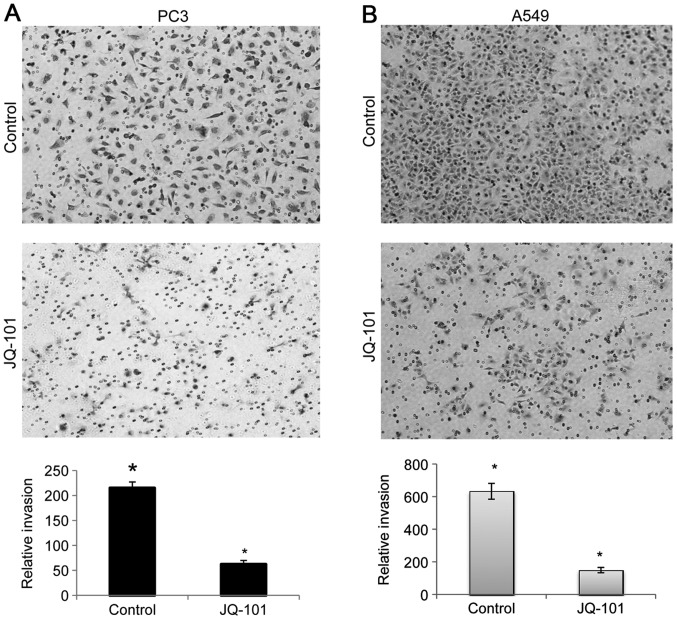
JQ-101 decreases cancer cell invasion. A total of 2.5×10^4^ (A) PC3 or (B) A549 cells in 500 μl medium mixed with JQ-101 (50 μM) or vehicle were loaded onto transwells coated with matrigel. After 48-h incubation at 37°C, the transwells were removed from 24-well plates and stained. The invading cells were quantitated by light microscopy. A representative experiment from three independent experiments is shown. Asterisks indicate significant differences between two groups (^*^p<0.05).

Recent studies have shown that SIRT1 plays an important role in cancer cell migration and invasion (14,16,46). We therefore determined whether SIRT1 inhibition by JQ-101 could inhibit cancer cell invasion. PC3 prostate cancer and A549 lung cancer cells, which exhibit invasive behavior, were exposed to JQ-101, and the effects on cancer cell invasion were quantitated, using a Transwell chamber assay system with matrigel coated on the top of an ECM-like membrane to prevent the transmigration of non-invasive cells. Exposure to JQ-101 significantly decreased invasion compared to vehicle control, producing a 3.5-fold reduction in PC3 cell invasion and a 4.2-fold reduction in A549 cells (Fig. 6A and B), suggesting that JQ-01 exerts an inhibitory effect on cancer cell invasion.

## Discussion

SIRT1 has attracted much attention for its functional roles in cancer (47). We report here a new SIRT1 inhibitor JQ-101, a compound possessing a polyprenylated acylphloroglucinol (PPAP) core, which exhibits selective inhibition of tumor cell line growth and survival though induction of apoptosis and cellular senescence, and significantly suppresses cancer cell invasion.

To date, several SIRT1 inhibitors have been developed with a broad range of potency in SIRT1 inhibition (32–36). The IC_50_ for JQ-101 against STIR1 is approximately 30 μM, a potency similar to several of the previously reported SIRT1-inhibitory compounds, including sirtinol (31), cambinol (32), salermide (37), tenovin-6 (34) and splitomycin (35). Most of the SIRT1 inhibitors identified previously also possess SIRT2 inhibitory activity, due to the high level of sequence similarity between SIRT1 and SIRT2. While JQ-101 also exhibited inhibitory effects on SIRT2, 5-fold higher concentrations were necessary, making JQ-101 somewhat SIRT1-specific. Our results show that JQ-101 can inhibit the growth and survival of a panel of cancer cell lines. Although we cannot rule out the possibility that minor inhibitory effects of SIRT 2 activity may contribute in part to the observed selective cytotoxicity of JQ-101, these studies were carried out under conditions far below the IC_50_ for SIRT2. In addition, our results show that SIRT1 knockdown greatly impairs the effect of JQ-101 on cell growth and survival (Fig. 4D), demonstrating that SIRT1 is the major target of JQ-101.

It has been shown that SIRT1 inhibition can induce apoptosis in many cancer cell types, in particular p53-wild-type cells (45,48–50); alternatively, SIRT1 inhibition can also induce cell senescence (51–53). Our results show that JQ-101 can trigger either apoptosis in p53-wild-type LNCaP cells or induce cell senescence in p53-null H1299 cells (Fig. 5). Depending upon the specific genetic background of the cancer cell, SIRT1 inhibition by JQ-101 may utilize different downstream targets of SIRT1 to induce either apoptosis or cell senescence, to suppress cancer cell growth and survival.

We also show in this study that JQ-101 treatment can greatly reduce cancer cell invasion (Fig. 6). We have previously identified SIRT1 as a positive regulator of epithelial-to-mesenchymal transition (EMT), cell invasion, and metastatic growth of prostate cancer cells, and put forward SIRT1 as a potential therapeutic target to reverse EMT and to prevent prostate cancer progression (8,19). Consistent with our findings, it has been shown that SIRT1 inhibition reduces cancer cell migration and invasion while SIRT1 activation promotes cancer metastasis (12,15,23,54). These results suggest that SIRT1 plays an important role in cancer invasion and metastasis, and that development of SIRT1 inhibitors may represent a critical new targeted strategy to prevent cancer metastasis and progression.

Given our findings that JQ-101 can selectively suppress cancer cell growth by inducing tumor cell apoptosis and senescence, and significantly inhibit cancer cell invasion, these results suggest a potential role of JQ-101 or a derivative in a SIRT1-targeted approach to cancer therapy. Studies are under way to delineate the effects of JQ-101 on tumor formation, growth and metastasis in mouse models *in vivo*. JQ-101 will also serve as a novel chemical scaffold for future development of more potent SIRT1 inhibitors to be used in cancer treatment.

## Figures and Tables

**Table I:** Cytotoxicity measurement of JQ-101 in multiple cancer/normal cell lines.

Cancer cell lines	IC_50_ (μM)
LNCaP	20
Jurkat	25
H460	30
Ramos	33
A549	40
MDA-MB-231	60
ZR75	66
H1299	85
PC3	119
Normal cell lines	
PZ-HPV-7	250
MRC5	300
